# The residency levels' effect on pediatric dental rehabilitation operation time

**DOI:** 10.1186/s12909-023-05009-2

**Published:** 2024-01-08

**Authors:** Raniah Baakdah, Shahad Al-kharouby, Shrouq Al-Sharif, Rabab Al-Nakhli, Yara Al-Sulami, Raghad Al-Qarni, Mohammed Yasir Al-Hindi

**Affiliations:** 1 Department of Dental Services - King Abdulaziz Medical City (KAMC), Ministry of National Guard-Health Affairs (MNGHA), Jeddah, Saudi Arabia; 2 College of Dentistry Umm Al-Qura University, Makkah, Saudi Arabia; 3 College of Dentistry King, Abdulaziz University, Jeddah, Saudi Arabia; 4 College of Dentistry King, Khalid University, Abha, Saudi Arabia; 5 Department of Pediatrics, King Abdulaziz Medical City, Jeddah, Saudi Arabia; 6 Research Office, King Abdullah International Medical Research Center (KAIMRC), Ministry of National Guard-Health Affairs (MNGHA), Jeddah, Saudi Arabia; 7 College of Medicine, King Saud Bin Abdulaziz University for Health Sciences, (KSAU-HS), Jeddah, Saudi Arabia

**Keywords:** General anesthesia, Pediatric dental rehabilitation, Operation time, Pediatric dental resident, Senior vs. junior, Dental procedure

## Abstract

**Background:**

Postgraduate pediatric dental residents' competency, to perform dental rehabilitation procedures under General anesthesia (GA), at different levels of training is challenging for operation time control. An adequate operation time (OT) for children decreases morbidity risk and improves hospital time utilization efficiency. The aim of the study is to assess the effect of pediatric dental resident training level on OT for pediatric dental rehabilitation procedures under GA at King Abdulaziz Medical City (KAMC).

**Methods:**

A cross-sectional study included pediatric dental rehabilitation performed under GA by pediatric dental residents at (KAMC) -Jeddah from October/2015 to September/2022. The primary outcome was OT, and the predictive variable was resident training levels. A linear regression analysis was used to compare OT between procedures performed by junior (years 1–2) or senior (years 3–4) trainees, adjusting for patient and operative factors.

**Results:**

One thousand seven pediatric dental rehabilitation cases were performed under GA by junior (13) and senior (31) residents. The univariant analysis indicated that OT for senior residents was significantly longer (13 min) than for junior residents. However, the linear regression analysis showed that senior residents had a significantly shorter OT when considering the more dental procedures performed per case under GA than junior residents. Senior residents took significantly more radiographs and performed more primary pulp therapies and multi-surface anterior colored restorations under GA than junior residents.

**Conclusions:**

The OT for pediatric dental rehabilitation procedures under GA is associated with resident training level. The total OT was significantly longer based on procedure number, type, and resident level. The study indicated that senior residents could manage more complex cases in a shorter time. The finding emphasizes the importance of assigning GA cases to residents based on their level and the case's complexity. Additionally, it helps standardize the resident privileges under GA and understand the impact of residency training on hospital efficiency.

## Background

General anesthesia (GA) is the most frequently used pharmacological intervention in pediatric dental care and the most preferred type of analgesia among parents [1–3]. GA is considered safe in treating pediatric dental caries, but the risk and complications reported intra- and postoperatively should be considered [4–7]. The risk of postoperative morbidity following pediatric dental rehabilitation procedures under GA ranges from negligible to more than 90% [8–10]. Common complications of GA are postoperative nausea and vomiting, hypothermia, laryngospasm or bronchospasm and pulmonary edema [4]. Dental practitioners must be aware of GA risks and the factors that trigger the development of complications, such as patient age, medical status, dental needs, staff experience, premedication use, anesthesia time, intubation, and medication type [5, 11–15].

The anesthesia time is the period from the start to the end of an anesthesia service [16]. Pediatric dental rehabilitation timing has been previously studied as a factor for hospital efficiency in the form of planned time or actual start and finish times; type of operator (attending or. resident); turnover and patient transport; and percentage of cancelled surgeries to report OT utilization and productivity [17–20]. GA timing can be affected by the ease of induction, complexity of the dental procedures, skill and level of the anesthetist, and efficiency of the time-in and time-out protocols [21]. The GA time is one of the factors reported to trigger the development of complications [21–23]. The control of the operation time has been studied and reported to decrease the incidence of morbidities and improve resource utilization efficiency [20]. On the other hand, the extended anesthesia time significantly increases the risks of complications and mortality when it exceeds 1 h to 6 h [24–26]. The duration of dental rehabilitation procedures at multiple centers in KSA was reported to range from 10 to 295 min. Foley’s study showed that the duration of GA did not influence the incidence of complications and reported that operator experience did not influence the procedure length (experienced provider vs. a provider-in-training) [27]. The main factors influencing OT were patient age and medical status, dental treatment type, number of teeth treated, and presence of a dental resident [20].

Postgraduate pediatric dental residents must have the basic skills to participate in treating dental rehabilitation cases under GA. Working under general anesthesia is a task that can be entrusted to residents at different supervision levels, which are “Only allowed to observe”: (no resident work), "Direct supervision": (resident work as co-activity with the supervisor or work independently under the supervision of the supervisor), and “Indirect supervision”: (resident work under indirect supervision). For board certification, the progress of the resident’s technical skill based on the number of completed cases and OT are quantitatively assessed continuously [28]. Daily booked GA cases are assigned to residents based on the supervising consultant’s opinion regarding the patients’ treatment needs and the resident’s level of experience. By evaluating the resident’s skills and speed, the OT could accurately be estimated for efficient utilization of hospital services. Although not all residents at the same level have the same competency skills, some pediatric dentistry programs still consider the resident level the prime factor for residents' GA services privilege. The determination of resident OT and its relation to patient and procedure factors under GA has never been studied in Saudi pediatric dentistry post-graduate programs. To our knowledge, no previous study, powered enough in the literature has compared the OT of pediatric dental rehabilitation procedures under GA between different groups of pediatric dental residents, especially in Saudi Arabia. Our null hypothesis stated no difference in OT between different-level residents. The aims of this study are to determine OT for different levels of pediatric dental residents at KAMC and to assess its relationship with the patient variables and the treatment procedures.

## Methods

The KAIMRC (King Abdullah International Medical Research Centre) Internal Review Board approved this cross-sectional chart review and granted an exemption from requiring informed consent (IRB/1514/22). The study reviewed all pediatric dental cases treated under GA at KAMC from October/2015 to September/2022. Patients who were > 16 years of age, underwent combined surgeries, were placed under deep sedation and operated on by nonresident operators, and had missing data were excluded. Electronic dental and medical records from KAMC Hospital were accessed to retrieve dental and anesthesia records. The following variables were extracted: age; sex; ASA classification, occlusion type (primary, mixed, permanent) treating resident level R (1,2,3,4); surgeon operation time OT (The time recorded from the start of the dentist work by drilling/incision to end of the dentist work and announcement of throat pack removal to transfer the patient for the anesthesia team care); operation date; number of intraoperative radiographs; total number of treated teeth; number of treated sextant; total number of dental procedures; and treatment type (restorative, extraction, both). The dental procedures include root canal therapy (permanent tooth) or pulp therapy (primary tooth); stainless steel crown; sealants; extraction; restoration type, site and number of surfaces restored (one-surface posterior restoration, multi-surface posterior restoration, one-surface anterior restoration, multi-surface anterior restoration); and surgery. For analysis, residents (R) were grouped based on level of training into senior (R3 and R4) and junior (R1 and R2) levels.

Sample size calculation using online software [29], comparing two independent means. Based on previous literature [20], the average operator time required per case was 76 min (± 37). A consensus among pediatric dental consultants at KAMC is that the expected difference of 45 min will be considered significant. Also, we accounted for the unequal sizes of junior to senior staff of a ratio 1:2.5. Therefore, assuming a pooled standard deviation of 37 min, the study would require a sample size of 11 for the junior group and 24 for the senior group (i.e., a total sample size of 32) to achieve a power of 80% and a level of significance of 5% (two-sided), for detecting a true difference in means between the test and the reference group of 45 min.

Data were analyzed using IBM SPSS (Statistical Package for the Social Sciences) version 28 (Arkon, NY, IBM Corp). For descriptive analysis, frequencies, percentages, and bar charts were used for categorical data. For measured data, means and standard deviations (SD) were normally distributed, while medians and 50% Interquartile Ranges (IQR) were used for skewed data. For univariate analysis, categorical variables were compared using Chi-square or Fisher’s exact tests depending on the numbers of the outcomes. While measured data were compared using t-tests for normally distributed data or Mann–Whitney for skewed data. To compare means of OT among the four levels of residency, One Way Analysis of Variance (ANOVA) was used. The level of significance was predetermined at P < 0.05. To examine for independent factors, a model was created where the dependent variable was the OT, and the independent variables were operator Level (senior Vs. junior), operator gender (male Vs female), and the chosen operative factors that were statistically or clinically significant. A linear regression analysis was used.

## Results

Thirty-five pediatric dental residents were trained at KAMC-Jed for formal academic years between October 2015 to September 2022. There were 13 male residents and 22 female residents. As some residents were trained at KAMC for more than one level, the final involved resident sample was 13 junior residents and 31 senior residents (Table 1). Cases performed under GA were 25 (2%) by R1 residents, 324 (32%) by R2 residents, 298 (30%) by R3 residents and 360 (36%) by R4 residents.

**Table 1:** Pediatric dental resident characteristics at KAMC-Jed 2015–2022

Total Pediatric Dental Resident Number		35
**Gender**	Male	13
	Female	22
**GA rotation**	One Residency Level	18
	Multiple Residency Levels	17
**Resident Level**	Senior (R3 and R4)	31
	Junior (R1 and R2)	13

About 1807 pediatric dental rehabilitation cases performed under GA from October/2015- to September/2022 were reviewed in the study. Dental sedation cases (23), patients operated by a nonresident trainee (5) or by a certified specialist/consultant (704) or had missing data (68) were excluded. Out of 1007 cases, the age of the children treated under GA ranged from 1 to 15 years, with a median of 5 years. Approximately 68% of treated patients were six or younger. Eighty percent of children were healthy (ASA I), 17% had controlled medical diseases (ASA II), and 3% had complicated medical conditions. Fifty-six percent of treated children had primary dentition, 34% had mixed dentition and 10% had full permanent dentition. Residents took intraoperative radiographs of 352 patients (35%). The number of radiographs that were taken ranged from 1 to 8, and the median number of radiographs taken was 6 (133 cases, 38%).

**Fig. 1:**
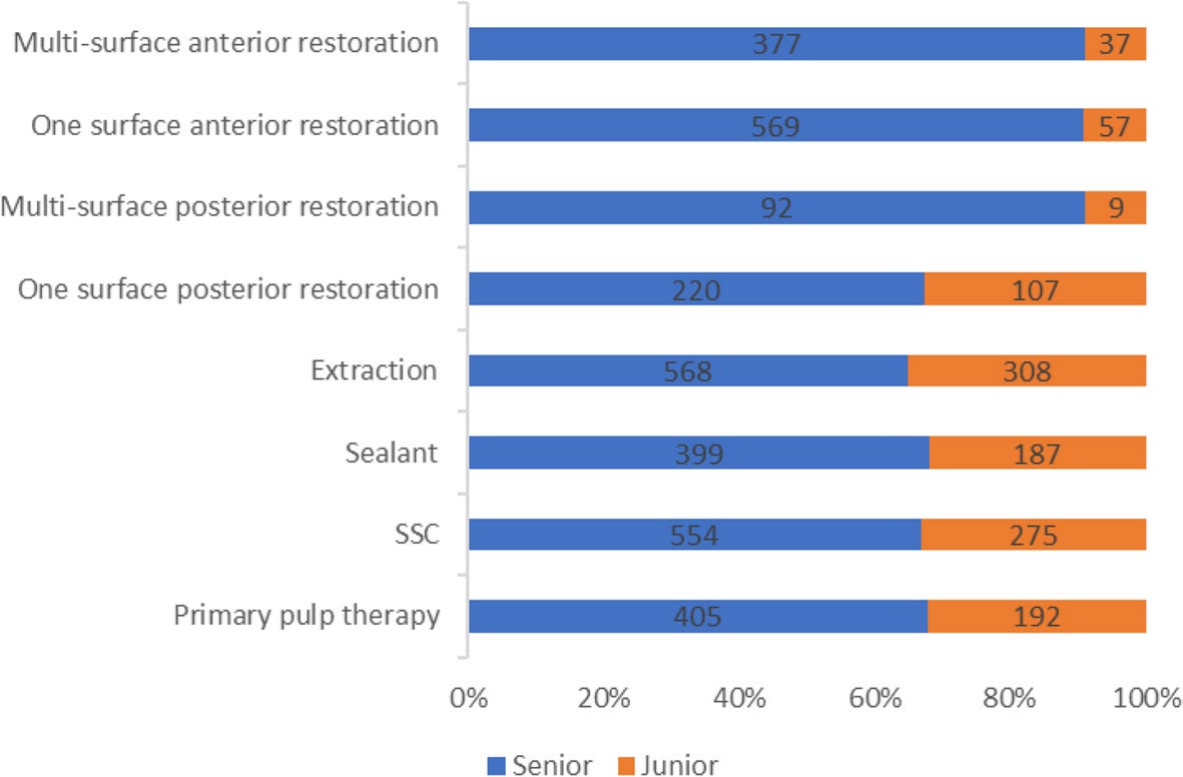
The frequency of dental procedures performed under GA by residents

The number of treated teeth per case ranged from 1 to 24, with a median of 13 teeth. Eleven to 20 teeth were treated in 80% of patients. The total number of dental procedures performed per case ranged from 1 to 32. The median number of procedures performed was 16. Only 5% of the patients underwent fewer than 10 procedures, and most (82%) involved 10–20 procedures. The treatment involved 5 or 6 sextants of the oral cavity in 85% of the patients. The teeth were treated by different dental procedures, ranging (0–24) extraction, (0–13) colored restoration, (0–12) sealant, (0–11) SSC, and (0–9) pulp therapy. The frequency of dental procedures performed by residents is illustrated in Fig. 1.

**Fig. 2:**
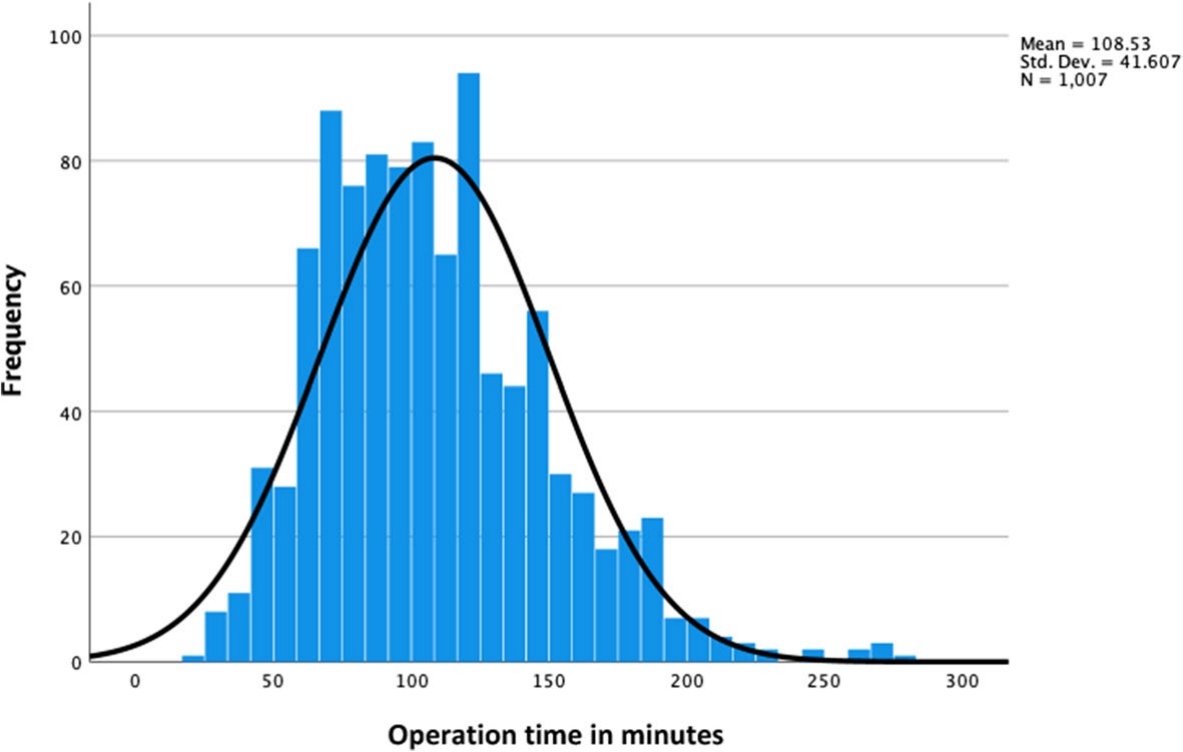
Operation time for dental rehabilitation cases treated by Pediatric dental residents

The OT for residents ranged from 20 to 280 min, with a median of 102 min and a mean of 108.5 min (± 41.6) (Fig. 2). The cases OT was 1 h or less for 10%, 2 h for 56%, and 3 h for 28%, and only 6% of residents needed more than 3 h of OT. Male residents treated 210 (± 21) patients, with a mean OT of 115 min (± 42), and female residents treated 797 (± 77) patients, with a mean OT of 106 min (± 41) (*P* = 0.927). The analysis of unadjusted OT for different residency levels revealed that OT increased with a higher residency level (*P* < 0.001) (Table 2). Moreover, the result was significant, indicating that senior residents had a higher median unadjusted OT (113 ± 42.9 min) than that of juniors (100 ± 37.5 min) (*P* < 0.001).

**Table 2:** Analyses for pediatric dental rehabilitation under GA based on residency level

Resident level	Number of treated cases (%)	Mean OT (± SD)	*P* (value)
R1	25 (2%)	97.04 (± 38)	< 0.001*
R2	324 (32%)	100.33 (± 37.5)	
R3	298 (30%)	112.87 (± 45)	
R4	360 (36%)	113.11 (± 41)	

*ANOVA

For univariate analysis, residents were categorized into Senior (R3&4) and Juniors (R1&2). Table 3 showed no significant difference in age or medical status of the patients treated under GA by junior and senior residents. The dentition type was significantly related to resident level. Senior residents mostly treated patients with primary and mixed dentition (359 and 240 cases sequentially), and junior residents mostly treated patients with permanent dentition (44 patients), with a *P* = 0.05. Statistically, there was no difference in the total number of treated teeth, but the number of dental procedures performed was significantly higher among senior resident’s median (IQR) 16 (13–19) vs. 15 (13–18) (*P* < 0.001). The number of dental sextant treatments was similar between senior and junior residents. The seniors took radiographs in 24% of cases for documentation (mostly 6 radiographs), while juniors requested radiographs in 11% of the cases (mostly 3 radiographs).

**Table 3:** Clinical and procedural factors that affect OT based on residency level

*Variables*	Senior	Junior	*P*(value)
***Operation time (OT)***	113 (± 43)	100 (± 38)	< 0.001^b^*
***Patient Age***	5.7 (± 2)	5.8 (± 2)	0.07^£^
***Patient Health***			
Healthy	513 (79%)	263 (81%)	0.302ω
Medically compromised	136 (21%)	63 (19%)	
***Patient occlusion***			
Primary	359 (55%)	200 (57%)	0.05^¥^
Mixed	240 (36%)	105 (30%)	
Permanent	59 (9%)	44 (13%)	
***Treatment type***			
Extraction	8 (1%)	11 (3%)	0.051ω
Restoration	92 (14%)	39 (11%)	
Combined	558 (85%)	299 (86%)	
***Total treated teeth***	13 (9–16)	13 (9–15)	0.186 α
***Total dental procedure***	16 (13–19)	15 (13–18)	< 0.001 α*
***Sextants involved***	5 (5–6)	5 (5–6)	0.18 α
***Radiograph***	0 (0–3)	0 (0–2)	0.006 α*
**Posterior restorations**			
One-surface	0 (0–1)	0 (0–1)	0.304 α
Multi-surfaces	0 (0–0)	0 (0–0)	0.170 α
**Anterior restorations**			
One-surface	1 (0–2)	1 (0–2)	0.536 α
Multi-surfaces	0 (0–2)	0 (0–1)	0.008 α*
***Sealants***	2 (0–4)	1 (0–4)	0.106 α
***Primary pulp therapy***	1 (0–3)	1 (0–2)	0.045 α*
***Stainless steel crown***	4 (2–6)	4 (1–6)	0.195 α
***Extraction***	4 (2–7)	4 (2–7)	0.663 α
***Oral Surgery***			
No	651 (99%)	344 (99%)	0.691ω
Yes	6 (1%)	5 (1%)	

¥ Fissure exact test

£ t-test

α Mann–Whitney test

ω Chi-square test

b Independent sample t-test

* Significant *P* value < 0.05

The total number and general type of treatments were not different between senior and junior residents, but junior residents approximately performed more extractions. Statistically, senior residents significantly performed more primary pulp therapies and multi-surface anterior colored restorations than junior residents under GA. The study reported low numbers of permanent pulp therapies, surgeries, and ready-made space maintainer procedures. The included surgery during dental rehabilitation were operculectomy and mesiodens removal.

In Table 4, a linear regression model was built to assess the predictors for OT adjusting for resident level (seniors) and gender (male). The following variables were built into the model based on the significance level in the univariate analysis (treatment type, total number of dental procedures, multi-surface anterior restorations, primary pulp therapy). The adjusted OT was significantly and independently shorter in seniors and males. The total number of procedures, multi-surface anterior restoration and primary pulp therapy significantly and independently predicted a longer OT, even after considering other variables. Case treatment type did not predict OT.

**Table 4:** Linear regression analysis for factors affecting the OT of pediatric dental rehabilitation procedures

Constant	Beta	*P* (Value)
Operator Level (Senior Vs. Junior)	-0.080	0.003*
Operator Gender (Male Vs Female)	-0.055	0.044*
Treatment type	0.012	0.670
Total number of dental procedures	0.237	< 0.001*
Multi-surface anterior restorations	0.235	< 0.001*
Primary pulp therapy	0.226	< 0.001*

^*^ Significant *P* value < 0.05

## Discussion

The study found that the involvement of residents in procedures performed under GA increased as the residency level increased. The low level of junior residents' involvement is explained by their lack of experience, limited clinical skill and uncertain decision-making, which are crucial for operators performing procedures under GA. However, juniors were allowed to perform procedures under GA for selected cases as KAMC consultant was continuously available during the operation for direct supervision.

Our study reported a mean OT of 108.5 (± 42) minutes for different-level residents at KAMC-Jed. Most patients were treated within 2 h, as recommended by the FDA, to avoid the risks associated with GA [30]. According to the literature, the mean duration of dental GA was 78 min in Germany, 145 min in Spain, 55 min in the USA, 60 min in the UK, and 124 min in Saudi Arabia [4, 31–33]. Forsyth’s study reported that most pediatric dental rehabilitation procedures performed under general anesthesia (73%) at Seattle Children’s Hospital/USA were finished early or on time compared to the planned/booked OT [20]. Saudi Pediatric dental residents' OT was better than the previously reported timing of Saudi pediatric dentist OT [4].

The study rejected the null hypothesis and found that OT was significantly associated with resident level. Initially, the univariate analysis showed that senior-level residents reported significantly longer OTs. The average 13-min OT difference between senior and junior may not be clinically significant duration. It could be explained by the longer time needed for senior cases with self-critical assessment, definitive diagnosis, skillful treatment, and frequent interruptions with indirect supervision during dental procedures compared to juniors who had a close supervision help to educate juniors resident closely and ensure the quality of treatment. Foley and Soldani’s study [27] indicated that the seniority skill effect on OT should be respected when comparing consultants/specialists with residents. Additionally, the basic residents’ competency development (speeds and skill) increases with experience and level [23, 34]. The study's linear regression analysis showed that the senior resident level was a significant predictor for a shorter OT, accounting for other variables in the model that represent resident demographics and the complexity of the procedures.

Furthermore, the study found that pediatric dental resident gender is a significant predictor, as male pediatric dental residents more frequently report a shorter OT. Although such findings have never been reported before among pediatric dentists, an explanation for gender-based differences in previous general surgery resident studies indicated that females had less operative autonomy and less confidence than males [34, 35].

Most of the patients treated in the study were young and healthy, similar to previous studies [36, 37]. Our study did not consider pediatric patient age or medical condition as significant predictors for OT among different resident levels. The literature reported that health status and age were significant predictors of treatment type under GA [38, 39]. Results that were published on the effects of age and medical status on OT were contradictory. Forsyth reported that surgeons were more likely to complete procedures on older, medically compromised patients in a shorter time [20], but Yi et al. [23] found that OT was significantly longer in older, medically compromised patients. In our study, there was no significant difference in the age or medical status of the patients treated under GA by junior and senior residents. However, older, healthy patients were more often treated by junior residents.

The study found that patient occlusion type and treatment modality were not statistically significant for residents’ OT. Other studies reported that extractions had a significantly shorter OT, and the treatment of more teeth had a significantly longer OT [20]. Our study reported that juniors operated more frequently on patients with permanent dentition and mostly performed extractions than seniors. The total number of treated teeth and sextants involved was not significantly different among the residents; however, the total number of procedures performed and radiographs taken was significantly higher among senior residents. Most dental procedures were not different among the residents, but seniors performed significantly more anterior multi-surface restorations and primary pulp therapies, which had significantly longer OTs. This study showed that seniors’ cases and procedures to be performed under GA were more complex. The low numbers of permanent pulp therapies, surgeries, and ready-made space maintainer procedures were similar to previous reports [2, 37, 40].

The implications of the present study are to balance priorities between the education of pediatric dental residents, patient safety, and efficient management of facilities and resources. The limitations of this study include its retrospective, single-institution design, which may limit the applicability of the results to other institutions. This study may be subject to selection bias, as the supervisor assigned cases based on the resident skill. However, there was no evidence to suggest selection bias between resident levels or genders. Differences in level and gender may be due to resident cohort effect, but this was mitigated by seven years follow-up for four levels of resident data and by controlling for case treatment factors in the multivariate analysis. Further multi-center study is needed to recommend GA cases assignment to resident skill level and formulate a statistical template to calculate OT based on the number and type of procedures. Further work is required to identify causes for skills gender-based disparities to determine strategies that optimize all residents' training.

## Conclusions

The present study examined a local institution (KAMC) database over seven years to assess the effect of resident level on the operation times of pediatric dental rehabilitation procedures under GA. Resident level and gender, with procedure number and type, had a significant impact on total OT. This study added a practical evidence for the development of OT competency by the progression of pediatric dentistry residency training. It determine the dental procedures that significantly increases cases complexity and the treatment time required by residents.

## Data Availability

The datasets used and analyzed during the current study are available from the corresponding author on reasonable request. Most data generated or analyzed during this study are included in this published article (tables and figures).

## Abbreviations

KAMC: King Abdulaziz Medical City
KAIMRC: King Abdullah International Medical Research Centre
IRB: Institutional Review Boards
GA: General anesthesia
OT: Operation time
R: Resident level
FDA: Food and Drug Administration
